# Catheterism in the Female

**Published:** 1859-07

**Authors:** Robert Battey

**Affiliations:** Rome, Georgia


### ARTICLE IV.

*Catheterism in the Female.* By ROBERT BATTEY, M. D., of Rome, Georgia.

There is perhaps no minor operation in the whole catalogue, in which expertness in the operator is more highly appreciated by the patient, and a blundering awkwardness so seldom forgiven, as catheterism in the female. To perform this little office acceptably, two things are necessary, 1st—A full acquaintance with the anatomy of the parts to be operated upon, and secondly, an educated sense of touch, which will enable the practitioner to determine definitely the structure with which it comes in contact, without resorting to that most humiliating expedient—aiding the hand by the use of the eye. A convenient and successful mode of accomplishing the service, is the following:

The operator, having washed his hands, whether clean or otherwise, by way of assurance to the patient upon this point, as also to soften them, and render the sense of touch more acute, takes his position in a chair to the left of the patient, who should be upon her back, conveniently near to the edge of the bed. He now anoints the catheter with oil or lard, using the index finger of the left hand, holding the instrument in his right, between the thumb and middle finger, with the end of the index closing the mouth of the catheter—raising the cover a little, he passes both hands under it, the left to the vulva, beneath the flexed thigh of the patient, the right to the symphysis pubis. Introducing the left index into the vagina, with its palp upon the urethra, he draws the point of the finger along the under surface of the canal, until it drops off at the end—the meatus urinarius, which he thus locates definitely in his mind—now passing the finger back into the vagina, and resting it upon the whole length of the urethra, he places the point of the catheter against the finger as a guide, slides it down gently into the meatus, and through the urethra into the bladder, fully sensible the while, of the correctness of the manœuvre, by the impression made upon the finger in the vagina. If the first attempt should be unsuccessful he withdraws the instrument and tries again; using more care to keep the point in the median line. In this and other manifestations in and about the vagina, the sense of touch may be greatly aided by closing the eyes, and concentrating the mind fully upon the business in hand.

If a small vessel, as a cup or bowl, be used for receiving the urine, it should be held with the finger, dipping down a little into it, that it may be known when it is nearly full, and to be emptied. A more readily obtainable, and perhaps, preferable recipient, is a porter or wine bottle, which should be surrounded by a napkin, and emptied when thought to be half full. A much neater and more convenient apparatus used by the writer, consists of a piece, say three or four feet long, of elastic tube, of goose-quill size, adjusted over the mouth of the catheter before its introduction, allowing the free extremity to hang over the side of the bed, and dip into the usual chamber vessel always at hand. It will be evident, that the elastic tube forms a syphon which rapidly empties the bladder; and when the catheter is removed it empties itself perfectly into the vessel, without danger of soiling either the person or bedding of the patient; and, also, that it obviates all necessity for any special receptacle for the urine, as well as any special dressing to protect the bed. Happily the ease and convenience with which the apparatus is cleansed, removes all objections under this head. After washing the hands with soap, raise the tube over the basin and let it gradually down into the water, catheter foremost. When quite filled with water, close the free end of the tube with the finger, and hang it over the edge of the vessel. Our syphon is again in action, and will continue to discharge fresh portions of clean water so long as we may choose to add it—until all doubt as to the entire cleanliness of the tube is removed.

The catheter should never be roughly introduced or withdrawn—no amount of force is required in either case—gentleness and patience will always be more successful. In removing the instrument, if difficulty be encountered, rotate the catheter a little, and make a steady but very gentle traction upon it; engage the patient in conversation; try to draw her mind off from the catheter to some more pleasing subject, at the same time quieting her apprehensions as to your ability to remove it when you see proper. If all other means fail, allow it to remain until the urine has accumulated and partially distended the bladder—when it is probable the removal will be easily effected.

If the patient be hysterical, it is desirable that we should not resort to the catheter, if to be avoided, lest we thereby subject ourselves to the necessity of repeating the operation twice daily, for many days. When indispensable in these cases, and in others, where the bladder is greatly distended, it is well that the catheter be of such size as to fill completely the urethral canal, for it occasionally happens with a small instrument, the sphincter suddenly gives way and the bed is flooded with urine, which escapes more rapidly around the catheter than through it, often leaving upon the mind of our patient, in her disgust, the impression, that she is indebted for the accident to carelessness in receiving the discharge.

